# Dynamic Localization of Glucokinase and Its Regulatory Protein in Hypothalamic Tanycytes

**DOI:** 10.1371/journal.pone.0094035

**Published:** 2014-04-16

**Authors:** Magdiel Salgado, Estefanía Tarifeño-Saldivia, Patricio Ordenes, Carola Millán, María José Yañez, Paula Llanos, Marcos Villagra, Roberto Elizondo-Vega, Fernando Martínez, Francisco Nualart, Elena Uribe, María de los Angeles García-Robles

**Affiliations:** 1 Departamento de Biología Celular, Facultad de Ciencias Biológicas, Universidad de Concepción, Concepción, Chile; 2 Departamento de Bioquímica y Biología Molecular, Facultad de Ciencias Biológicas, Universidad de Concepción, Concepción, Chile; 3 Facultad de Artes Liberales, Universidad Adolfo Ibañez, Viña del Mar, Chile; Hosptial Infantil Universitario Niño Jesús, CIBEROBN, Spain

## Abstract

Glucokinase (GK), the hexokinase involved in glucose sensing in pancreatic β cells, is also expressed in hypothalamic tanycytes, which cover the ventricular walls of the basal hypothalamus and are implicated in an indirect control of neuronal activity by glucose. Previously, we demonstrated that GK was preferentially localized in tanycyte nuclei in euglycemic rats, which has been reported in hepatocytes and is suggestive of the presence of the GK regulatory protein, GKRP. In the present study, GK intracellular localization in hypothalamic and hepatic tissues of the same rats under several glycemic conditions was compared using confocal microscopy and Western blot analysis. In the hypothalamus, increased GK nuclear localization was observed in hyperglycemic conditions; however, it was primarily localized in the cytoplasm in hepatic tissue under the same conditions. Both GK and GKRP were next cloned from primary cultures of tanycytes. Expression of GK by *Escherichia coli* revealed a functional cooperative protein with a S_0.5_ of 10 mM. GKRP, expressed in *Saccharomyces cerevisiae*, inhibited GK activity *in vitro* with a K_i_ 0.2 µM. We also demonstrated increased nuclear reactivity of both GK and GKRP in response to high glucose concentrations in tanycyte cultures. These data were confirmed using Western blot analysis of nuclear extracts. Results indicate that GK undergoes short-term regulation by nuclear compartmentalization. Thus, in tanycytes, GK can act as a molecular switch to arrest cellular responses to increased glucose.

## Introduction

Specific brain regions, such as the hypothalamus and brain stem, are able to detect and respond to changes in glucose concentration, triggering neuroendocrine responses that regulate feeding behavior [1]. In the hypothalamus, specifically in the arcuate nucleus (AN), neurons are capable of responding to changes in glucose and lactate concentrations [2], [3]. AN neurons are in close contact with ependymal cells, known as tanycytes, which are the main glial cell present in the basal hypothalamus [4], [5]. Their proximal pole is located within the ventricular wall, and their long cellular process is projected towards the ventromedial hypothalamus [6], [7].

There are four types of tanycytes: α1, α2, β1 and β2 [8]. α2 and β1 tanycytes are localized in the lower lateral wall of the third ventricle; they have extended cell processes that contact the neurons in the AN, in particular neuropeptide Y (NPY) neurons [5], [9], [10] and pro-opiomelanocortin (POMC) neurons [11]. β2 tanycytes form the cerebrospinal fluid (CSF)-median eminence (ME) barrier, and their extended processes contact vessels devoid of the blood–brain barrier and are sometimes in direct contact with microvessels present in the ME [12]. We have demonstrated that these cells express proteins, including glucose transporter (GLUT2), glucokinase (GK) and ATP-sensitive K^+^ (K^+^_ATP_) channels, involved in the β cell glucose sensing mechanism in the pancreas, indicating their possible involvement in hypothalamus-mediated glucosensing [6], [13]. Notably, GLUT2 is located in the proximal pole of tanycytes, which are in direct contact with the CSF in the third ventricle [6]. GK was localized mainly in the tanycyte nuclei in the euglycemic condition [13]. We have also demonstrated that glucose induces a rise in intracellular Ca^2+^ in tanycyte cultures, which depended on ATP generated by the uptake and metabolism of glucose [14]. Independent study in brain slices has shown that selective stimulation of tanycyte cell bodies by glucose and also non-metabolizable analogs of glucose evokes robust ATP-mediated Ca^2+^ responses [15]. Nevertheless, both studies showed that Ca^2+^ waves were dependent on ATP release and subsequent activation of P2Y1 receptors. In addition, intracerebroventricular infusion of ATP stimulates neurons localized in the lateral hypothalamic area [16], dorsomedial hypothalamic nucleus [17] and ventromedial nucleus VMN [18]. Similarly, we have demonstrated that tanycytes release lactate in response to glucose [19]. ATP or lactate release by tanycytes may modulate neuronal activity in hypothalamic areas associated with feeding behavior, including the arcuate nucleus (AN) and VMN, which are in close contact with these cells. Moreover, extracellular ATP and possibly ADP may regulate food intake via activation of P2Y1 receptors [20]. Lactate and its subsequent release to neighboring neurons through the monocarboxylate transporter (MCT) may lead to enhanced ATP synthesis, closure of K^+^_ATP_ channels, and neuronal depolarization. In this context, we demonstrated that tanycytes expressed MCT1 and MCT4, which are involved in the efflux of lactate [19]. Moreover, orexigenic and anorexigenic neurons of the AN have a high membrane immunoreactivity for MCT2, which is involved in lactate influx [11], suggesting that tanycytes are implicated in an indirect control of neuronal activity by glucose. Our previously results indicate that the response to glucose generated by tanycytes depends on the glycolytic metabolism and not oxidative phosphorylation [14]. In this context, we have shown that alloxan, a GK inhibitor, blocks calcium signaling in tanycytes.

GK or hexokinase IV (EC2.7.1.1) has a low affinity for phosphorylating glucose (S_0.5_ 10–15 mM), and is not inhibited by glucose-6-phosphate [21]. GK is expressed in the liver [22], pancreatic β cells [23], jejunal enterocytes, neuroendocrine cells and the brain [3], [24], [25], [26]. Although GK is a product of one gene, an alternative promoter is used in hepatic and pancreatic tissues, generating tissue-specific isoforms that differ in the first fifteen amino acids [27]. In rat hypothalamic tissue, the expression of the pancreatic isoform has been reported [3], [24], [26]. At the protein level, immunoblotting and enzyme assays have confirmed the presence of GK in rat and human hypothalamic extracts [26], [28], [29]. However other isoforms that are produced by alternative splicing have been described in the hypothalamus and pituitary but are not functional [26], [30], [31].

Hepatic GK activity is regulated at the post-translational level through binding to GK regulatory protein (GKRP), which functions as an anchor protein, modulating GK activity and mediating its nuclear translocation [32], [33]. Under basal glucose conditions (5–7 mM), hepatic GK is predominantly bound to GKRP within the hepatocyte nucleus in an inactive state [34]. After exposure to high glucose (10–30 mM), GK is released from GKRP and translocates to the cytoplasm [34], [35], [36], [37]. Immunocytochemical localization studies have demonstrated that GKRP is predominantly localized to the nucleus of hepatocytes [38]; however, the subcellular localization of GK varies according to the metabolic state of the cell, including glycogen deposition [39]. GKRP had an inhibitory effect over GK activity, displaying a K_i_ of 1 µM [40]. In pancreatic β cells, where GKRP is not expressed, GK is mainly localized in the extranuclear compartments with the highest activity in the cytoplasm [38], [41]. However, there is some controversy in the literature as to whether GKRP also regulates GK in pancreatic β cells [42], [43], [44]; the vast majority of studies report that is not expressed in rodent β cells [38], [41], [43], [44]. Tiedge et al. [45] confirmed the presence of an inhibitory factor for GK in the pancreas; however, Northern blot analysis revealed that it is not GKRP [45]. To our knowledge, there is only one study that reported the presence of an alternatively spliced GKRP variant expressed in the β cells of rodents [42]. In the hypothalamus using isotopic *in situ* hybridization, the same authors described GKRP expression in the periventricular nucleus. However, the hybridization signal was strongly suggestive of a positive reaction in glial periventricular cells. Therefore, subcellular GKRP-dependent GK translocation may regulate the enzymatic activity in tanycytes in accordance with the metabolic needs of the cell. Using immunolocalization, we demonstrated the presence of GK in tanycytes and in a minor proportion in periventricular neurons [13]. In euglycemic conditions, GK was detected largely in the tanycyte nuclei, suggesting short-term regulation by GKRP [13]. To determine if tanycyte GK is sequestered to the nuclear compartment in response to glucose, we compared its localization in hypothalamic and liver tissues of the same rats in response to hypoglycemic, normoglycemic and hyperglycemic conditions. These studies clearly demonstrated increased nuclear localization of tanycyte GK in a form opposite to that observed in hepatocytes. Because these results suggest the presence of GKRP in tanycytes, we cloned both GK and GKRP from highly enriched primary tanycyte cultures and the recombinant proteins showed a very high sequence identity with the pancreatic and hepatic proteins, respectively. *In vitro* kinetic analysis demonstrated GK activity and its inhibition by GKRP. In highly enriched primary tanycyte cultures, the intranuclear localization of GK and GKRP increased in the presence of high glucose concentrations, indicating that GK undergoes an intracellular translocation in tanycytes that could be attributed to the particular functions of these cells.

## Materials and Methods

### Animals

#### Ethics Statement

All animals were handled in strict accordance with the Animal Welfare Assurance (permit number 2010101A), and all animal work was approved by the appropriate Ethics and Animal Care and Use Committee of the Universidad de Concepcion, Chile. Adult male Sprague-Dawley rats between 200–300 g were used for the experiments. Animals were kept in a 12-h light/dark cycle with food and water *ad libitum*.

### Immunohistochemistry

The following antibodies and dilutions were used: rabbit anti-GK (1∶100, sc7908; Santa Cruz Biotechnology, Santa Cruz, CA, USA) and mouse anti-vimentin (1∶200, DAKO, Santa Barbara, CA, USA). The antibodies were diluted in a Tris-HCl buffer (pH 7.8) containing 8.4 mM sodium phosphate, 3.5 mM potassium phosphate, 120 mM sodium chloride, and 1% bovine serum albumin. Sections of the liver (20 µm) and frontal hypothalamus (40 µm) were fixed directly by immersion in 4% (w/v) paraformaldehyde for 12 h, incubated in 30% sucrose for 3 days and cut with a cryostat (Leica, microm HM520), and subsequently processed free-floating. Primary antibodies were incubated overnight at 22°C, and subsequently incubated for 2 h at 22°C with Cy^2^- Cy^3^-labeled secondary antibodies (1∶200; Jackson ImmunoResearch Laboratories, Inc., Pennsylvania, USA). The samples were counter-stained with the DNA stain, TOPRO-3 (1∶1000; Invitrogen, Rockville, MD, USA), and the slides were analyzed by confocal laser microscopy (Carl Zeiss, LSM700, Jena, Germany). A total of five rats were used per glycemic conditions.

### Immunocytochemistry

Cultured cells were grown on poly-L-lysine-coated (Sigma-Aldrich, St. Louis, MO, USA) glass cover slides in 24-well plates, fixed with 4% paraformaldehyde in PBS for 30 min, washed with Tris-HCl buffer (pH 7.8), and incubated in the same buffer containing 1% bovine serum albumin (BSA) and 0.2% Triton X-100 for 5 min at 22°C. Samples were then incubated with the following primary antibodies overnight at 22°C: rabbit anti-GK (1∶100, sc 7908; Santa Cruz Biotechnology), rabbit anti-GKRP (1∶100, sc-11416; Santa Cruz Biotechnology), mouse anti-vimentin (1∶200, Dako), rabbit anti-GFAP (1∶200, DAKO), mouse anti- MAP2 (1∶50, Chemicon Temecula, CA), mouse anti- neurofilaments (1∶1, Hybridoma Data Bank), goat anti- DARPP32 (1∶50, Santa Cruz Biotechnology), rabbit anti- GLUT2 (1∶100, Alpha Diagnostic International, San Antonio, TX), chicken anti-MCT1 (1∶100, Millipore, Temecula, CA), rabbit anti-MCT4 (1∶20, Millipore) and anti-Kir1.6 (1∶200, Santa Cruz Biotechnology). Cells were next incubated with Cy^2^- or Cy^3^-labeled secondary antibodies (Jackson ImmunoResearch Laboratories), counterstained with the DNA stain, TOPRO-3 (1∶1000, Invitrogen), and analyzed using confocal laser microscopy Carl Zeiss, LSM700).

### Image analysis

Digital images were analyzed using ImageJ image analysis software (National Institutes of Health, Bethesda, Maryland, USA, http://rsb.info.nih.gov/ij/). For nuclear quantification of GK and GKRP, color channels were separated, and the regions of interest (ROIs) were selected using the nuclear marker channel, by manually outlining the regions of interest using drawing tools included in the software. Data from 200-500 nuclei were combined, and the mean and standard deviation for each condition were obtained using GraphPad Prism 5.03 software (GraphPad Software Inc., San Diego CA, USA).

### Cell culture

Highly enriched primary tanycyte cultures from 1-day postnatal brains (10–12 rats) were isolated and immunocharacterized following the method described previously [6], [14], [19], [46]. Briefly, the hypothalamus was removed and further dissected to obtain a region close to the ependymal layer. The dissection was carried out with the samples submerged in dissection buffer containing 10 mM HEPES (pH 7.4, 340 mOsm/L). Trypsinized tissue was transferred to planting medium containing MEM, (Invitrogen) with 10% (v/v) fetal bovine serum (FBS) (Thermo Fisher Scientific Inc., Waltham, MA, USA), and 2 mg/mL DNAse I (Sigma-Aldrich). Cells were seeded at 1.2×10^5^ cells/cm^2^ in culture dishes treated with 0.2 mg/mL poly-L-lysine (Sigma-Aldrich). After 2 h, the culture medium was changed to MEM (5 mM glucose) supplemented with 10% FBS, 2 mM L-glutamine, 100 U/mL penicillin, 100 µg/mL streptomycin, and 2.5 µg/mL fungizone (Thermo Fisher Scientific, Inc). Cells were cultured in the same dish for 3 weeks, and the medium was changed every 2 days. To study the effect of glucose on the intracellular localization of GK and GKRP, cells were grown in glucose-free DMEM supplemented with 10% FBS for 6 h and were subsequently supplemented with 15 mM glucose for 30 before immunocytochemistry analyses.

### Reverse transcription-polymerase chain reaction

Total RNA was isolated from the hypothalamic area, highly enriched primary tanycyte cultures, liver and pancreatic tissue using Trizol (Invitrogen). For RT-PCR, 1 µg of RNA was incubated in 10 µL reaction volume containing 10 mM Tris-HCl (pH 8.3), 50 mM KCl, 5 mM MgCl_2_, 20 U RNase inhibitor, 1 mM dNTPs, 2.5 µM oligo d(T) primers, and 50 units of MuLV reverse transcriptase (New England Biolabs, Ipswich, MA, USA) for 10 min at 23°C followed by 30 min at 42°C and 5 min at 94°C. Parallel reactions were performed in the absence of reverse transcriptase to control for the presence of contaminant DNA. For amplification, a cDNA aliquot in a volume of 12.5 µL containing 20 mM Tris-HCl (pH 8.4), 50 mM KCl, 1.6 mM MgCl_2_, 0.4 mM dNTPs, 0.04 units of Taq DNA polymerase (Gibco-BRL, Carlsbad, CA, USA), and 0.4 mM primers was incubated at 94°C for 4 min, 94°C for 15–50 s, 55°C for 30–50 s, and 72°C for 30–35 s for 35 cycles. PCR products were separated by 1.2–1.5% agarose gel electrophoresis and visualized by staining with ethidium bromide. The following sets of primers were used for RT-PCR analysis: pancreatic GK (M58759.1 positions 123–978), sense 5’ AAT ACT CAA AAG CCA TCC CCA AGC C 3’ and antisense 5’ ATG CCC TTG TCT ATG TCG TCG TGC C 3’ (expected product of 856 bp); hepatic GK, sense (NM_012565; positions 1–388) 5’ ATG GCT ATG GAT ACT ACA AGG TGT G 3’ and antisense 5’ TGC ATT CAG AGA TGT AGT CAA AGA G 3’ (expected product of 388 bp); GKRP (X68497.1; positions 451–868), sense 5’ AGA CAG AAG ATA GCG CCC TAC ACG 3’ and antisense 5’ CTT TGA GAG GAC ACA ACA CCC TGG 3’ (expected product 418 bp); and β-actin, sense 5’ GCT GCT CGT CGA CAA CGG CTC and antisense 5’ CAA ACA TGA TCT GGG TCA TCT TCT C 3’ (expected product 353 bp).

### Cloning of GK and GKRP from tanycyte cultures

From highly enriched primary tanycyte cultures in MEM (5.5 mM glucose) for 14 days, total RNA was extracted using TRIzol Reagent (Invitrogen). For RT-PCR, 1 µg of total RNA was incubated in 20 µL of reaction buffer containing 50 mM Tris-HCL (pH 8.3), 50 mM KCl, 4 mM MgCl_2_, 10 mM DTT, 1 U RNAse inhibitor (Fermentas International Inc., Burlington, Ontario, Canada), 1 mM dNTPs and 2.5 µM oligo(dt) primer for 5 min at 37°C followed by 1 h at 42°C with 200 U reverse transcriptase (RevertAid™ H Minus M-MuLV, Fermentas) and 10 min at 70°C. Parallel reactions in the absence of reverse transcriptase were performed to control for genomic DNA contamination. The amplification reaction contained 20 ng of cDNA, 0.3 mM dNTPs, 1 mM MgSO4, 2.5 units of Platinum Pfx DNA polymerase (Invitrogen), 5 µL of the manufacturer’s amplification buffer, 0.5 nmol of specific primers and RNAse-free water up to a final volume of 50 µL. The following thermocycling conditions were used: 94°C for 3 min followed by 35 cycles at 94°C for 15 s, 60°C for 30 s and 68°C for 2 min. PCR products were separated and purified by 1.2% agarose gel. The follow primer sets were used to clone the complete coding region of GK and GKRP from tanycytes. GK (M25807.1): sense 5'- CAC GGT GCC CAT GGT GGA TGA CAG AGC CAG GAT GGA GG -3' antisense 5'- GCT GGG GAT CCT CAC TGG GCC AGC ATG CAA GC -3' (1398 bp). GKRP (X68497.1): sense 5′- CGA TCT ATC CAT GGC AGG CAC CAA AC -3′antisense 5′- CTG CAT TCT TGG ATC CTC AAT TCA GG -3′. The PCR products were subcloned into Invitrogen One Shot competent cells using a Zero Blunt TOPO PCR cloning kit (Invitrogen). Ligation was performed with 10 ng of the TOPO pCR vector, 10 ng of the PCR product, 200 mM NaCl and 10 mM MgCl2 incubated for 30 s at room temperature. Of this reaction, 4 µL was used to transform E.coli TOP10 competent cells by incubating them at 42°C for 30 s. Cells were grown in Agar-LB kanamycin (50 mg/µL) plates overnight at 37°C after which 5–10 colonies were selected to grow in LB/kanamycin medium to purify the plasmid. Plasmid purification was performed using the StrataPrep Plasmid Miniprep Kit (Stratagene), and insertion was verified by restriction assays using EcoRI. The plasmids were sequenced (Retrogen Inc, California USA) to determine the sequences of GK and GKRP in tanycytes.

### Expression and purification of recombinant GK and GKRP

The glial-GK cDNA was directionally cloned into the H_6_PQE-60 *E. coli* expression vector and expressed in *E. coli* JM 109. Expression induction was performed at 0.6–600 nm optical density using 0.5 mM isopropyl-β-D-thiogalactopyranoside (IPTG). The bacterial cells were disrupted by sonication on ice (5×30 s pulses) in a buffer containing 100 mM Tris-HCl (pH 7.5), 100 mM KCl, 5 mM DTT and protease inhibitors cocktail (Roche Applied Science, Indianapolis, In, USA). After centrifugation for 20 min at 96,000 ×g, the supernatant was applied to DEAE-cellulose column equilibrated with 10 mM Tris-HCl (pH 7.5). Proteins that were nonspecifically bound to the resin were removed by washing with the same buffer until the absorbance of the elutes at 280 nm dropped to approximately 0. The elution was performed by steps using the same buffer and increasing concentration of KCl (100, 200 and 300 mM), washing the resin until no protein was detected in each step. The recombinant GK was eluted with 200 mM KCl, and its purity was assessed by SDS-PAGE (12%). Western blot analysis was performed using a rabbit anti-GK antibody (1∶1000, Santa Cruz Biotechnology).

Glial-GKRP cDNA was directionally cloned into the pYES2.1/V5-His-TOPO yeast expression vector, and the protein was expressed in TRY104Δspe1 *Saccharomyces cerevisiae*. After the cells were transformed with 1 µg of vector carrying glial GKRP, they were cultured for 16 h at 30°C. Expression induction was performed with 0.11 M galactose overnight. The transformed cells were lysed mechanically and centrifuged for 20 min at 13,000 ×g, and the supernatant containing the protein slurry was applied to a DEAE-cellulose resin equilibrated with 10 mM Tris-HCl (pH 7.5), where the protein was retained. The unbound proteins were removed with 10 mM Tris-HCl and the proteins were eluted with 100, 200 and 300 mM KCl until no proteins was detected between each elution. The recombinant GKRP was eluted with 300 mM KCl, and its purity was assessed by SDS-PAGE (12%). Western blot analysis was performed using a rabbit anti-GKRP antibody (1∶1000, Santa Cruz Biotechnology, Inc).

### Enzyme assays

GK activity was determined as previously described with slight modifications [47]. A glucose-6-phosphate dehydrogenase-coupled reaction was used, and the activity was followed by measuring the increase in absorbance at 340 nm after 5 min incubation at 37°C. The reaction mixture consisted of 200 mM Tris-HCl buffer (pH 7.5), 2 mM MgCl_2_, 1 mM DTT, 1 mM ATP, 0.5 mM NADP^+^, 10 mM glucose, and 1 U/mL of glucose-6-phosphate dehydrogenase (Sigma-Aldrich). For kinetic constant determination, we used 5 µg/µL of purified GK in the reaction mixture. The S_0.5_ and n_H_ values of GK for glucose was obtained from a Hill plot of three saturation curves, and the Prism software was used for data analysis (GraphPad, Inc).

*In vitro* inhibition assays- In order to obtain an IC_50_ value for GKRP with respect to GK activity, the rate of the reaction in the presence of 0.05, 0.1, 0.15 and 0.25 µM of purified GKRP was evaluated in triplicate. The K_i_ value of GK/GKRP interaction was obtained from the graphs proposed by Segel [48], for use when an allosteric modulator stabilizes the T state of a cooperative enzyme, as is the case for GK [49], [50].

### Glycemic conditions induced by glucose injection

Rats were kept in the usual condition at 22°C, on a light/dark cycle of 12-h light and 12-h dark with *ad libitum* access to food and water. Only rats that had normal blood glucose (7.5 mM) were selected for GK translocation experiments. Rats that were fasted for 16 h, reaching 4–5 mM blood glucose levels, subsequently received an intraperitoneal (i.p.) injection with 2.3 mL of glucose (4 or 0.5 g/kg body weight) or saline to generate hyperglycemic, normoglycemic, and fasting conditions, respectively. Blood glucose levels in samples obtained from the tail vein as well as CSF from the third ventricle of cannulated rats were measured using the Accu chek kit. After 30 min, the rats were anesthetized using ketamine-xilazine (90 mg/Kg-10 mg/Kg) for subsequent sacrifice using decapitation. Livers and brains were harvested and fixed in 4% PFA for 24 h at 4°C, for immunohistochemistry analysis; nuclear protein extracts were also obtained from these tissues.

### Immunoblotting

Total protein extracts were obtained from rat liver, pancreas, periventricular hypothalamus, and tanycyte cultures and by homogenizing the tissue or cells in buffer A (0.3 mM sucrose, 3 mM DTT, 1 mM EDTA, 100 µg/mL PMSF, 2 µg/mL pepstatin A, 2 µg/mL leucopeptin, and 2 µg/mL aprotinin). The periventricular hypothalamus was obtained from fresh ice cold brains by making two transverse cuts, one at the optic chiasm and another just before the mammillary bodies, dissecting the area closest to the diencephalic third ventricle. Subsequently, the samples were sonicated on ice at 300 W (Sonics & Material INC, VCF1, Connecticut, USA) 3 times for 10 s. After centrifugation at 4000 ×g for 10 min, proteins were resolved by SDS-PAGE (50 µg/lane) in a 12% (w/v) polyacrylamide gel, transferred to PVDF membranes (0.45 µm pore, Amersham Pharmacia Biotech., Piscataway, NJ, USA), and probed with rabbit anti-GK, anti-GKRP, anti-lamin B1 and anti-β-actin antibodies. After extensive washing, the PVDF membranes were incubated for 1 h at 4°C with peroxidase-labeled anti-rabbit IgG (1∶5000; Jackson Immuno Research). The reaction was developed using the enhanced chemiluminescence (ECL) Western blotting analysis system (Amersham Biosciences). Negative controls consisted of incubating the membrane with a pre-absorbed antibody (anti-GK 1∶1000 with 100 mg/mL inductor peptide incubated at 4°C overnight), or the absent anti-GKRP.

### Nuclear extract preparation

To analyze the subcellular localization of GK *in situ*, hypothalamic nuclear extracts were prepared using the following protocol. Unicellular suspensions were obtained from the dissected tissues by trypsinization followed by disruption in ice cold PBS with protease inhibitors, DNAseI, 3 mM DTT and 1 mM EDTA. Cells were collected after centrifugation at 700 ×g for 5 min, and resuspended in ice cold lysis buffer for 10 min. After centrifugation at 850 ×g for 1 min, the cell pellets were resuspended in hypotonic buffer (10 mM HEPES, 1.5 mM MgCl_2_, 10 mM KCl, pH 7.9). The nuclei were isolated by centrifugation at 4600 ×g (Micromax RF centrifugue, IEC) for 1 min, and the nuclear pellets were resuspended in extraction buffer (20 mM HEPES, 1.5 mM MgCl_2_, 420 mM KCl, 0.2 mM EDTA, 20% glycerol, pH 7.9) and agitated on an orbital shaker at a maximum speed at 4°C for 45 min followed by centrifugation at 9300 ×g for 5 min. Nuclear extract aliquots were frozen in liquid nitrogen and stored at −80°C. To obtain nuclear extracts from cultured tanycytes, we used NE-PER Nuclear and Cytoplasmic Extraction Reagents (Thermo Scientific, Waltham, MA) following the manufacturer’s instructions. All procedures following the cell disruption were performed on ice or at 4°C. The purity of the nuclear extracts was confirmed by Western blot analysis using anti-lamin B1 antibody (ab16048, Abcam, Cambridge, England, UK), a nuclear marker.

### Glucorrachia and glycemia measures

Rats that were previously cannulated into the third ventricle in the bregma coordinates (Bregma AP 23.14 mm, ML 0.0 mm, DV 9.2 mm) were used. After one week of recovery, rats were fasted for 16 h and then were anesthetized by i.p. injection of ketamine-xilazine (90 mg/Kg-10 mg/Kg). Glycemia and glycorrhachia were measured at 15, 30 and 45 min after injection of glucose (4 g/kg body weight) using the Accu-chek kit from 2 µL of blood or CSF. For both parameters, time zero corresponds to measurements taken from fasted animals after injection of the same volume of saline (stress control). To calculate glucose levels in the CSF using the Accu-chek kit, which was developed for measuring blood glucose levels, it was necessary to perform a calibration curve using 1-20 mM glucose in artificial CSF.

#### Statistical analysis

The *in situ* studies were analyzed using one way ANOVA-Dunnet’s multiple comparison test. A total of 500 cells were quantified in five animals per condition. For immunocytochemistry analyses, a t-test with Welch correction was made in a total of 200 tanycytes per condition.

## Results

### Intracellular localization of GK in the hypothalamus and liver in response to glycemia

In order to establish the levels of cerebral glucose reached in the hyperglycemic condition, glycorrhachia was measured in third ventricle canulated rats. After one week of recovery, rats were fasted for 16 h after which glycemia (Fig. S1A) and glycorrhachia (Fig. S1B) were measured using the Accu-chek kit and 2 µL of blood or CSF. After injecting glucose (4 g/kg body weight, ∼ 2.3 mL), blood glucose levels reached a maximum of 25.5±6 mM at 30 min (Fig. S1A), and glycorrhachia reached a maximum level of 9.5±2.9 mM at 45 min (Fig. S1B). As shown in Fig. S1 (0 min), rats in the fasting condition and injected with 2.3 mL saline had blood glucose levels of 4.5±0.95 mM and glycorrhachia of 2.1±0.48 mM, values that are slightly higher than those obtained without injecting saline (3.2±0.83 mM and 1.6±0.28 mM, respectively).

**Figure 1:**
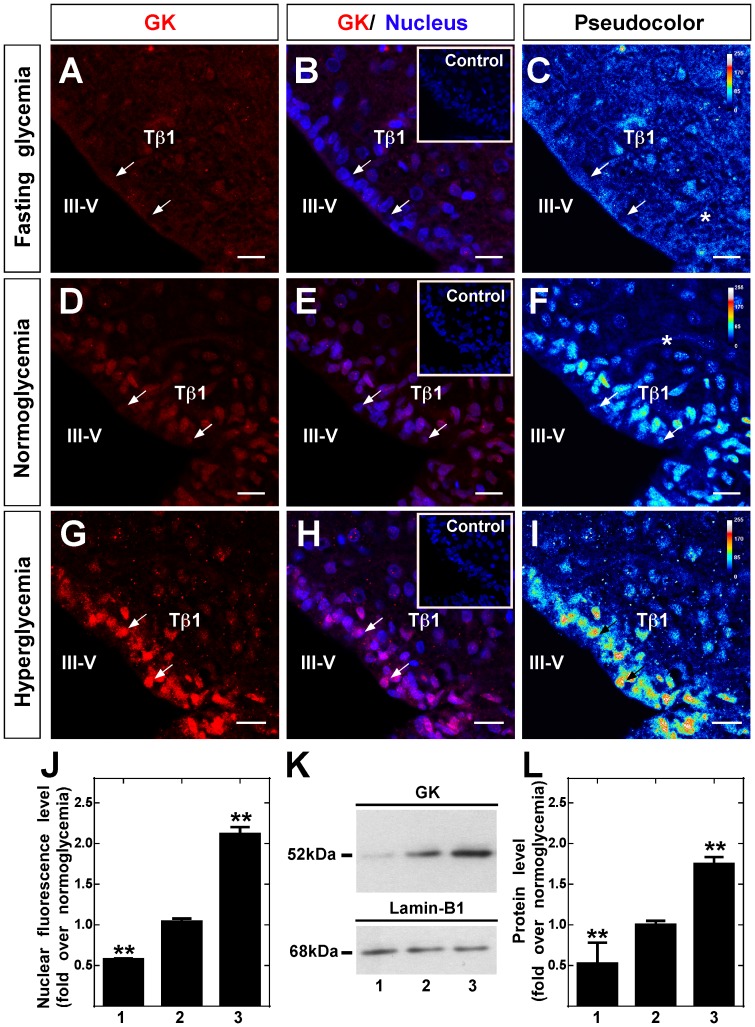
Immunolocalization and Western blot analysis of hypothalamic GK. **A-I**, Frontal sections of rat hypothalamus were obtained from animals exposed to different glycemic conditions and were immunostained with rabbit anti-GK (1∶100). Fasting (A–C), normoglycemia (D–F) and hyperglycemia (G–I). **C**, **F** and **I**, semi-quantitative analysis of GK immunofluorescence by pseudocolor (blue, negative signal; yellow, medium signal; red, high signal). Inserts in B, E and H correspond to the immunoreaction using anti-GK preabsorbed with inductor peptides. **J**, Quantitative analysis of nuclear intensity of tanycyte GK in different glycemic conditions. Statistical analysis was performed using the one way ANOVA-Dunnet test; *P*-values<0.05 were considered significant. Data represent the average fluorescence intensity of GK-positive nuclei in a total of 200 tanycytes obtained from five animals per condition. **K**, Immunoblots of GK (52 kDa, upper-panel) and lamin-B1 (68 kDa, lower panel) in nuclear protein extracts obtained in different glycemic conditions. **L**, Quantitative analysis of GK nuclear expression relative to lamin-B1. The nuclear localization of GK increased gradually in response to glycemia. Statistical analysis was performed using the one way ANOVA-Dunnet test; *P*-values<0.05 were considered significant. J-L, 1-3 represent fasting, normoglycemic and hyperglycemic conditions, respectively. Data represent the means±SD from six independent determinations. III-V: third ventricle; Tβ1: β1 tanycytes. Scale bars, 25 µm.

The liver and hypothalamus were obtained from the same animals to analyze GK localization in response to fasting condition, normoglycemia and hyperglycemia induced by 16 h fasting or 30 min post-intraperitoneal injection of 2.3 mL of saline or 0.5 g/kg or 4 g/kg body weight glucose, respectively. Hypothalamic and hepatic tissues of five rats were used per condition for immunohistochemical analysis and six rats for Western blot assays. Using high magnification images, GK immunolocalization (red) and nuclei (blue) were analyzed in frontal sections of hypothalamic tissue. The effect produced by glycemic conditions was evaluated semi-quantitatively by applying the pseudocolor function (Fig. 1C, F, and I). In hypoglycemia (4.5±0.7 mM), diffuse GK immunoreaction was mainly localized in the ventricular area (apical region of tanycytes) and in parenchyma of the AN (Fig. 1A–C). Because tanycytes have long cellular processes, GK displayed a low but superior immunoreaction to the negative control (Fig 1B, inset), suggesting a mainly cytoplasmic localization. In normoglycemia (7.2±1.1 mM), intense GK nuclear localization was detected in tanycytes (Fig. 1D–F). In hyperglycemia (24.6±1.6 mM), increased GK nuclear localization was detected (Fig. 1 G–I), which becomes more evident with colocalization with the nuclear marker (Fig. 1H) or using the pseudocolor representation (Fig.1I). Quantitative nuclear immunoreaction analysis revealed increased GK fluorescence intensity from the fasting to hyperglycemic condition (Fig. 1J). A lower immunoreaction was also detected in the tanycyte cytosol as is shown in the pseudocolor images (Fig. 1C, F and I, asterisks). Moreover, a representative Western blot image of nuclear GK protein levels in response to each glycemic condition is shown in Fig. 1K. A single band of 52 kDa, corresponding to GK was obtained (Fig. 1K, upper panel). Lamin B1 was used as a loading control for the nuclear fractions (Fig. 1K, lower panel). Quantification of the nuclear GK band normalized to the lamin B1 band was performed using Quantity one software. Data normalized to normoglycemic condition show a 2-fold reduction of GK protein level in hypoglycemic rats and a 2-fold increase in hyperglycemic rats (Fig. 1L).

**Figure 2:**
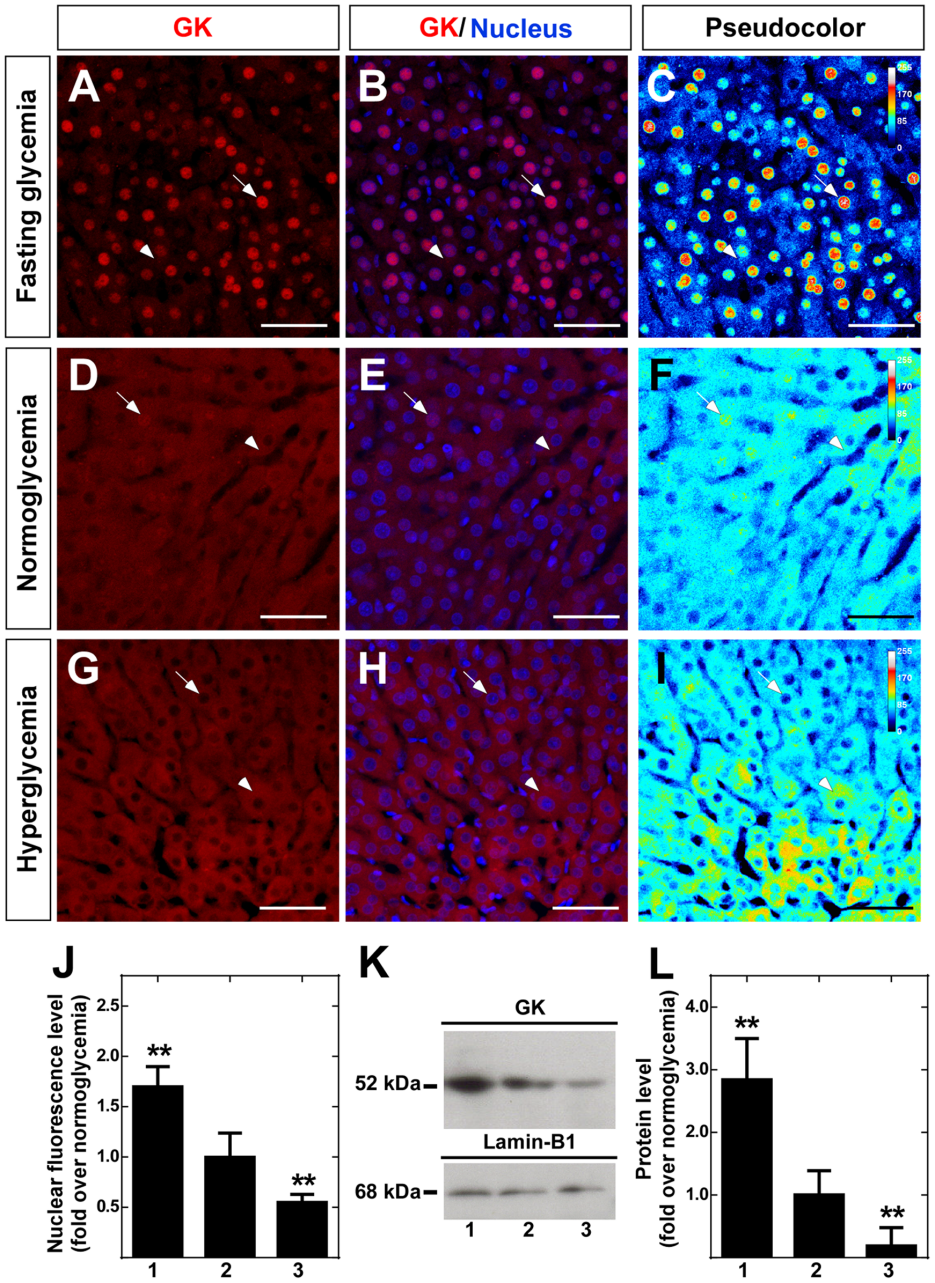
Immunolocalization and Western blot analysis of liver GK. **A-I**, Sections of liver were obtained from animals exposed to different glycemic conditions and were immunostained with rabbit anti-GK (1∶100). Hypoglycemia (A–C), normoglycemia (D–F) and hyperglycemia (G–I). **C**, **F** and **I**, semi-quantitative analysis of GK immunofluorescence by pseudocolor (blue, negative signal; yellow, medium signal; red, high signal). **J**, Quantitative analysis of nuclear intensity of hepatocyte GK at different glycemic conditions. Statistical analysis was performed using the one way ANOVA-Dunnet test; *P*-values<0.05 were considered significant. Data represent the average fluorescence intensity of GK-positive nuclei in a total of 200 hepatocytes. **K**, Immunoblots of GK (52 kDa, upper-panel) and lamin-B1 (68 kDa, lower panel) in nuclear protein extracts obtained in different glycemic conditions. **L**, Quantitative analysis of GK nuclear expression relative to lamin-B1. The nuclear localization of GK decreased gradually in response to glycemia. Statistical analysis was performed using the one way ANOVA-Dunnet test; *P*-values<0.05 were considered significant. J-L, 1-3 represent fasting, normoglycemic and hyperglycemic conditions, respectively. Data represent the means±SD from six independent determinations. Scale bars, 100 µm.

Similar immunohistochemical analysis was performed in hepatic tissue of the same rats (Fig. 2A–J). Because it has been previously reported that GK immunoreaction changes between different regions [51], an area close to perivenose region was selected. The pseudocolor results confirmed previously published data that GK nuclear localization was mainly observed in the hypoglycemic condition [34], [52], [53] (Fig. 2C, F and I). Quantitative analysis of this data normalized to values obtained in normoglycemia showed a 2-fold increase in nuclear intensity in response to hypoglycemia and a reduction in hyperglycemia (Fig. 2J). Western blot analysis of liver nuclear extracts of the same animals used in the hypothalamus assays (Fig. 1K) showed a 3-fold increase in nuclear GK level and weak levels in hyperglycemia (Fig. 2K-L). Thus, tanycyte GK is compartmentalized in an opposite manner to that observed for liver GK at 30 min of induced hyperglycemia.

### Hypothalamic tanycytes express the pancreatic GK isoform

Our results show that tanycyte GK undergoes a glycemia-dependent nuclear compartmentalization, which may indicate that GKRP is expressed in these cells. For this reason, we decided to evaluate whether GKRP is present in these cells and whether GK isoform expressed in tanycytes is the same as has been previously reported in the hypothalamus [26]. An extensive immunocharacterization of highly enriched primary tanycyte cultures was performed (Fig. S2), which demonstrated the expression of GLUT2, GK, MCT1, DARPP32 and KIR6.1 in these cells as well as the absence of neuronal marker expression, such as neurofilaments and MAP2. Similarly, we did not detect GFAP, an astrocyte marker that is expressed in α tanycytes [54]. Because β1- but not α-tanycytes express GK, MCT1, and MCT4 *in vivo* [13], [19], these results suggest a high enrichment in β1-tanycytes in these cultures.

**Figure 3:**
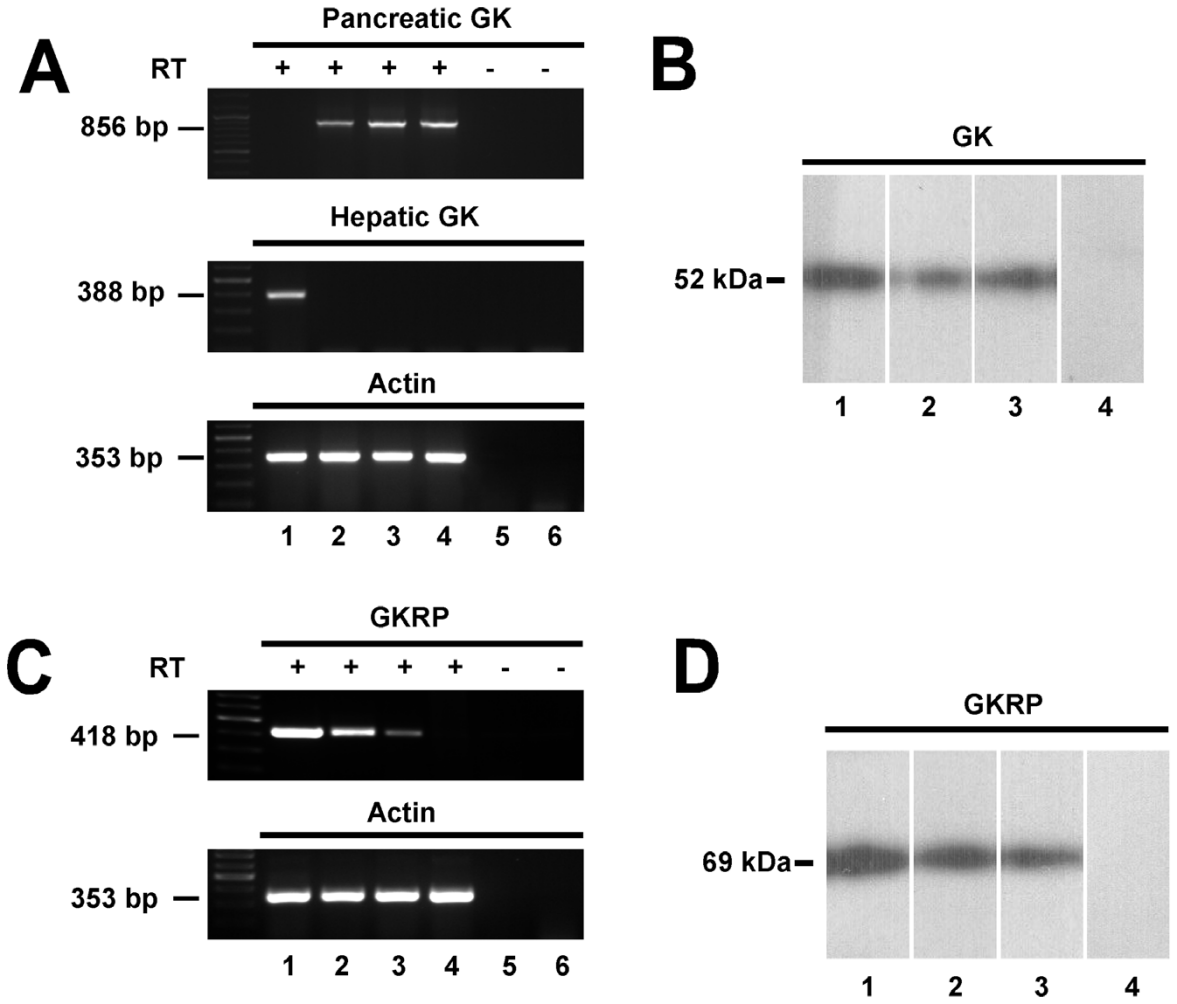
Identification of GK and GKRP isoforms expressed in tanycytes. **A**, GK sequences were amplified by RT-PCR using primers that were specific for pancreatic GK (856 bp; top panel), liver GK (388 bp; middle panel), and β-actin (353 bp; bottom panel). PCR products amplified from liver (lane 1), pancreas (lane 2), hypothalamus (lane 3), tanycytes in culture (lane 4) cDNA. Negative controls consisted of RT(-) of hypothalamus (lane 5) and tanycytes (lane 6). **B**, Immunoblots of GK in total protein extract from pancreas (lane 1), hypothalamus (lane 2) and tanycyte cultures (lane 3), negative control using pre-absorbed antibody (lane 4). **C**, RT-PCR to amplify GKRP (418 pb; upper-panel) and β-actin (353 bp; lower-panel) using liver (lane 1), hypothalamus (lane 2), tanycytes (lane 3) and pancreas (lane 4) cDNA. Negative controls consisted of RT(-) of hypothalamus (lane 5) and tanycytes (lane 6) cDNA. **D**, Immunoblots of GKRP in total protein extract from liver (lane 1), hypothalamus (lane 2) and tanycyte culture (lane 3), using the omission of anti-GKRP (lanes 4).

Using specific primers for pancreatic GK mRNA, a corresponding GK band was observed in samples obtained from the pancreas, hypothalamus and tanycytes cultures (Fig. 3A, lanes 2–4, upper panel). No amplification was detected in the liver samples (Fig. 3A, lane 1). Furthermore, using primers designed to specifically amplify liver GK, a band was only detected in the liver sample (Fig. 3A, lane 1, middle panel). No amplification product was observed in the hypothalamus and tanycyte cultures samples without reverse transcriptase (Fig. 3A, lanes 5–6). Bands of the expected size were obtained in all samples using primers specific for β-actin. (Fig. 3A, lanes 1–4, lower panel). These results suggest that cultured tanycytes maintain the expression of the pancreatic GK isoform.

GK expression in tanycyte cultures was also demonstrated using immunoblot analysis of protein extracts isolated from the pancreas, rat hypothalamus and tanycyte cultures (Fig. 3B, lanes 1–3). No band was detected in tanycyte culture protein extracts using anti-GK preabsorbed with inductor peptides (Fig. 3B, lane 4). Furthermore, GKRP expression in tanycyte cultures was analyzed using RT-PCR with primers specific for rat hepatic GKRP in hepatic, hypothalamic, tanycyte cultures and pancreatic samples. The GKRP band of 418 bp was apparent in samples from the liver, hypothalamus and tanycyte cultures (Fig. 3C, lanes 1–3, upper panel). Amplification product was not observed in pancreatic samples as well as hypothalamic and tanycyte samples amplified without reverse transcriptase (Fig. 3C, lanes 4–6, upper panel). Using Western blots analysis, the expression of GKRP in total protein extracts from tanycyte cultures was confirmed (Fig. 3D, lane 3); the same band of 69 kDa was obtained in liver and hypothalamic samples (Fig. 3D, lane 1–2). No band was detected when anti-GKRP was omitted in a total protein extract from tanycyte cultures. Therefore, RT-PCR and Western blots analyses confirmed the expression of GK and GKRP in tanycyte cultures.

**Figure 4:**
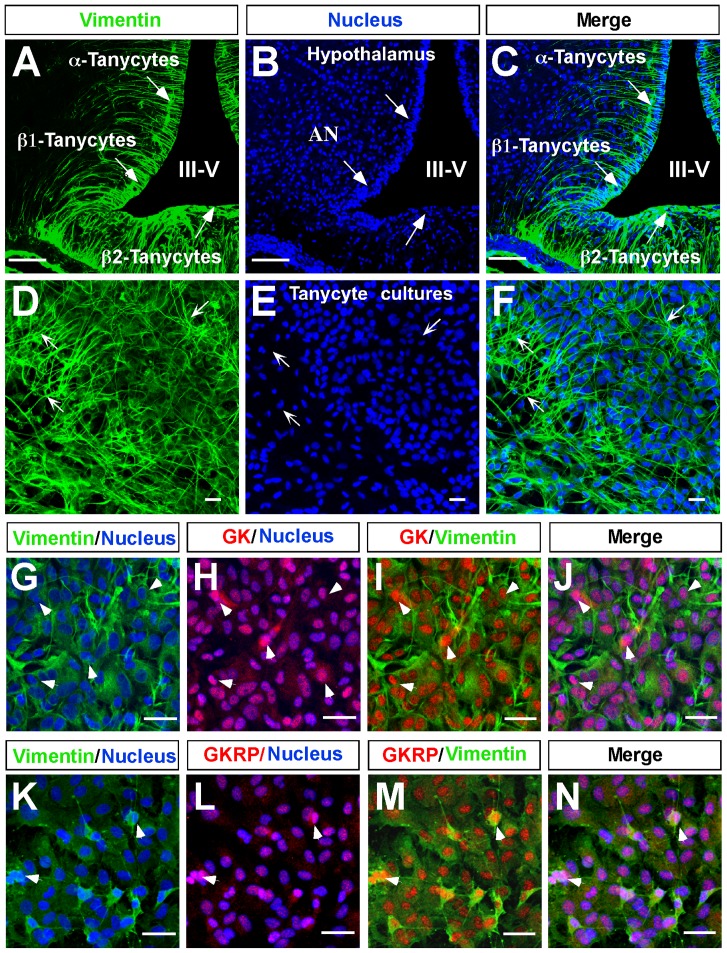
Hypothalamic tanycytes expressed GK and GKRP. **A–F**, Confocal microscopy analysis of immunofluorescence using anti-vimentin (green) and Topro-3 nuclear stain (blue). **A–C**, 40-μm slides of frontal rat hypothalamus showing vimentin expression in α and β-tanycytes. **D–F**, Tanycyte primary cultures, which maintain their polarization and vimentin expression. **G–J**, Immunodetection of GK in tanycytes cultured in 5. 5 mM glucose. **K–N**, Immunodetection of GKRP in tanycytes cultured in 5.5 mM glucose.

Immunocytochemical analysis revealed that tanycyte cultures maintain the intense reactivity to vimentin (Fig. 4D, arrows), showing a high number of cells organized into groups, with elongated cytoplasmic processes that maintain a degree of polarity (Fig. 4D–F). This morphology was very similar to that observed in the basal region of the hypothalamus (Fig. 4A–C). Immunocytochemical analysis using triple labeling revealed that GK is widely expressed in tanycytes cultured in 5.5 mM glucose (Fig. 4H–J), which was similar to that observed for vimentin (Fig. 4 G, I and J). Moreover, GK nuclear colocalization with the nuclear marker was detected in most cells (Fig. 4H and J, pink); however, a low colocalization with vimentin in very few cells was detected in the cytoplasm, which is most likely in the long extension of its cellular processes (Fig. 4H and J, arrowheads). A similar study was carried out with GKRP (Fig. 4K–N). GKRP was mainly localized in tanycyte nuclei; however, some GKRP was detected in the cytoplasm.

### GK and GKRP cloned from tanycyte cultures are functional

Because the tanycyte cultures exhibited the functional properties similar to *in situ* tanycytes, such as the capability of both ATP and lactate release in response to glucose [14], [19], and the immunocytochemical characterization confirmed a highly enriched tanycyte culture, we decided to clone GK (1398bp) and GKRP (1884 bp). Sequence analysis showed that tanycyte GK was identical to the pancreatic GK isoform (with one conserved amino acid substitution). In addition, tanycyte GKRP showed a 99% homology with hepatic GKRP (with three conserved amino acid substitutions and one non-conserved substitution). These nucleotide sequences were indexed in GenBank with the codes GCK (GK), KJ026953 and GCKR (GKRP), KJ026952.

**Figure 5:**
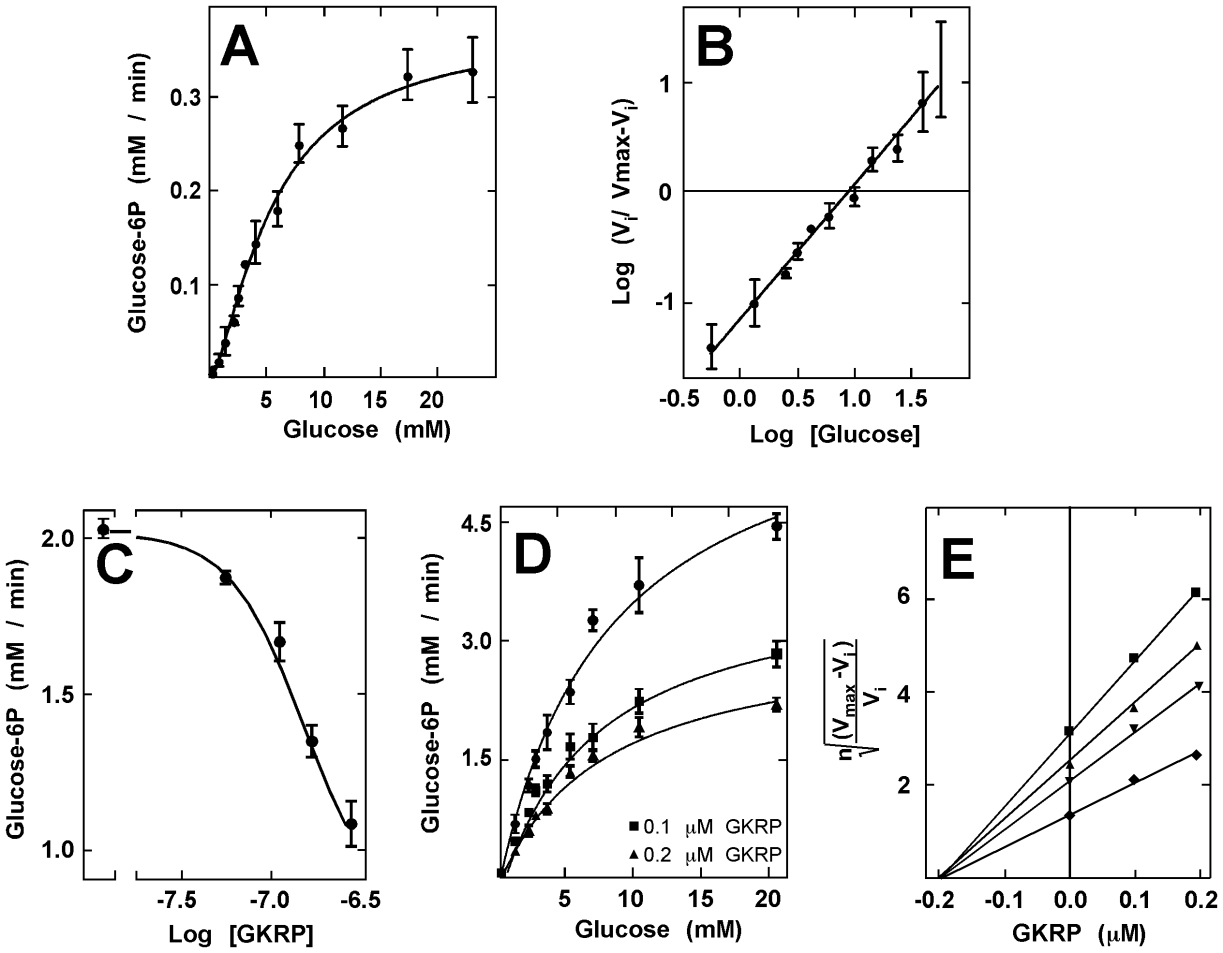
Kinetic characterization of GK and its inhibition by GKRP. **A**, Using a glucose-6-phosphate dehydrogenase-coupled method, we evaluated the concentration dependence of glucose phosphorylation by recombinant GK with 1 mM ATP and pH 7.5. **B**, Hill plot analysis of the data shown in A, displaying a positive cooperativity with a S_0.5_ of 10 mM and a 1.5 n_H_. **C**, Inhibitory effect of GKRP (0.05–0.25 µM) over GK activity. As has been reported in hepatocytes, GKRP inhibits glial GK in a dose-dependent manner, resulting in a median inhibitory concentration (IC_50_) of 0.28 mM. **D**, Effect of GKRP in GK saturation by glucose displaying a decrease in the maximal velocity of the reaction with 0.1 and 0.2 µM GKRP. **E**, Due to the cooperative behavior of GK, we performed a Segel-proposed plot of the data from D to determine the inhibition constant (K_i_) of GKRP upon GK. Results represent the mean±SD of three independent experiments.

Because recombinant GK and bacterial GK exhibit marked differences in their isoelectric points, recombinant GK was expressed in *E. coli,* and purified by ion exchange chromatography. Hexokinase activity was evaluated in the different fractions with 1 and 30 mM glucose, and the purity of the fractions was evaluated by Western blot analysis (data not shown). In order to obtain the catalytic constant of the recombinant GK, we performed saturation curves in a range of 0.5–20 mM glucose, maintaining the concentration of ATP at 1 mM. Dose-response studies performed up to 5 min revealed a cooperative behavior (Fig. 5A). The Hill plot revealed a S_0.5_ of 10±0.6 mM, which was close to that described for pancreatic GK [55]. The Hill coefficient (n_H_) was 1.5±0.07, indicating positive cooperativity (Fig. 5B). GKRP was expressed in *S. cerevisiae* and purified by ion exchange chromatography. The fractions with higher purity were selected by dot blot analysis using a PAGE-stained with Coomassie blue (data not shown). The i*n vitro* effect of GKRP on GK was determined by analyzing the IC_50_, which was obtained by assessing GK activity at 5 min in response to 10 mM glucose and increasing GKRP concentration. Given the yields obtained in the purification of GKRP, it was not possible to test higher concentrations of the protein; however by nonlinear extrapolation using the GraphPad Prism software 5.0, an IC_50_ value of 0.28 mM was obtained (Fig. 5C). In addition, GK saturation curves with glucose in the presence of increasing GKRP concentrations revealed a decrease in the V_max_ of the reaction, but not the S_0.5_ (Fig. 4D). Plotting the data using Segel’s graph for a cooperative enzymes showed a K_i_ of 0.21 µM (Fig. 4E).

### Nuclear translocation of GK in response to hypoglycemia in tanycyte cultures

**Figure 6:**
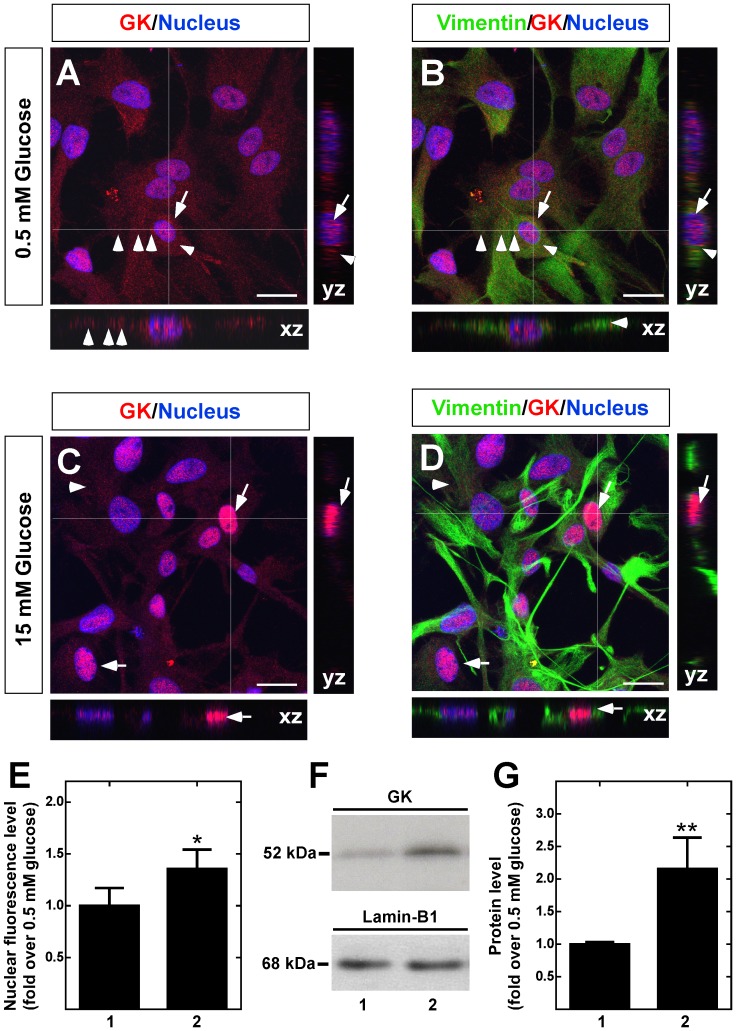
Dynamic localization of GK in cultured tanycytes in response to glucose. **A–D**. GK immunolocalization (red) in tanycytes preincubated with 0.5 mM glucose for 6 h (A–B) or incubated with 15 mM glucose for 30 min (C–D). Nuclei (A–C) and Vimentin (B, D) are stained in blue and green, respectively. Arrows and arrowheads show nuclear and cytoplasmic GK localization, respectively. Images were obtained by Z-stack reconstruction, and white lines indicate orthogonal planes (XZ and YZ) shown in the lateral and lower panels. **E**, Nuclear intensity of GK immunofluorescence in tanycytes cultured in the presence of 0.5 mM (lane 1) and 15 mM (lane 2) glucose. The results represent the mean±sd of 200 cells from three independent primary cultures. **F**, Immunoblots of GK (52 kDa, upper panel) and the nuclear marker, lamin-B1 (68 kDa, lower panel), in nuclear extracts obtained from cells cultured with 0.5 mM (lane 1) and 15 mM (lane 2) glucose. **G**, GK nuclear expression levels assessed by western-blot were quantified and normalized with lamin-B for cells cultured with 0.5 mM (lane 1) and 15 mM (lane 2) glucose. The data shows the means±SD from six independent experiments. * p<0.05; ** p<0.01. Scale bar in A–D, 50 µm.

In a manner similar to hepatic GK, GK cloned from tanycyte cultures has glucose phosphorylating activity, which is inhibited by GKRP *in vitro*. However, our data showed opposing changes in GK intranuclear localization in response to glucose in hypothalamic and hepatic tissues. For this reason, we decided to evaluate the intracellular localization of GK and GKRP in response to increasing extracellular glucose concentration in tanycyte cultures. As the glucose concentration in rats after 30 min of induced hyperglycemia reached a maximum of 8.15 mM (Fig. S1), we decided to evaluate a slightly higher dose, 15 mM, and compare it to baseline glucose, 0.5 mM. Tanycyte cultures were incubated for 6 h in 0.5 mM glucose as well as those immediately transferred to 15 mM glucose for 30 min and then processed for immunocytochemistry and Western blot analyses. Triple labeling immunocytochemistry using anti-GK or anti-GKRP in addition to anti-vimentin and Topro 3 as a nuclear marker was carried out, and images were processed by Z-stack reconstruction, and orthogonal planes were analyzed for nuclear and cytosolic distribution. Cells cultured in 0.5 mM glucose had nuclear GK immunoreaction and cytoplasmic localization, which was more evident in the orthogonal images (Fig. 6A, B, YZ and XZ frames). Cells incubated in 15 mM glucose had increased nuclear GK intensity (Fig. 6C, D), which can be attributed to a nucleocytoplasmic redistribution as judged by orthogonal frames (Fig. 6C, D, YZ and XZ frames). The weak signal detected in the cytoplasm may be due to the long cellular processes of these cells; thus, we decided to quantify the nuclear intensity of fluorescence as a result of the nuclear translocation, revealing a significant increase in GK nuclear intensity in response to 15 mM glucose (Fig. 6E). This result was confirmed using immunoblot analysis of nuclear extracts (Fig. 6F, G) and cytosolic extracts (Fig. S3A–B)

**Figure 7:**
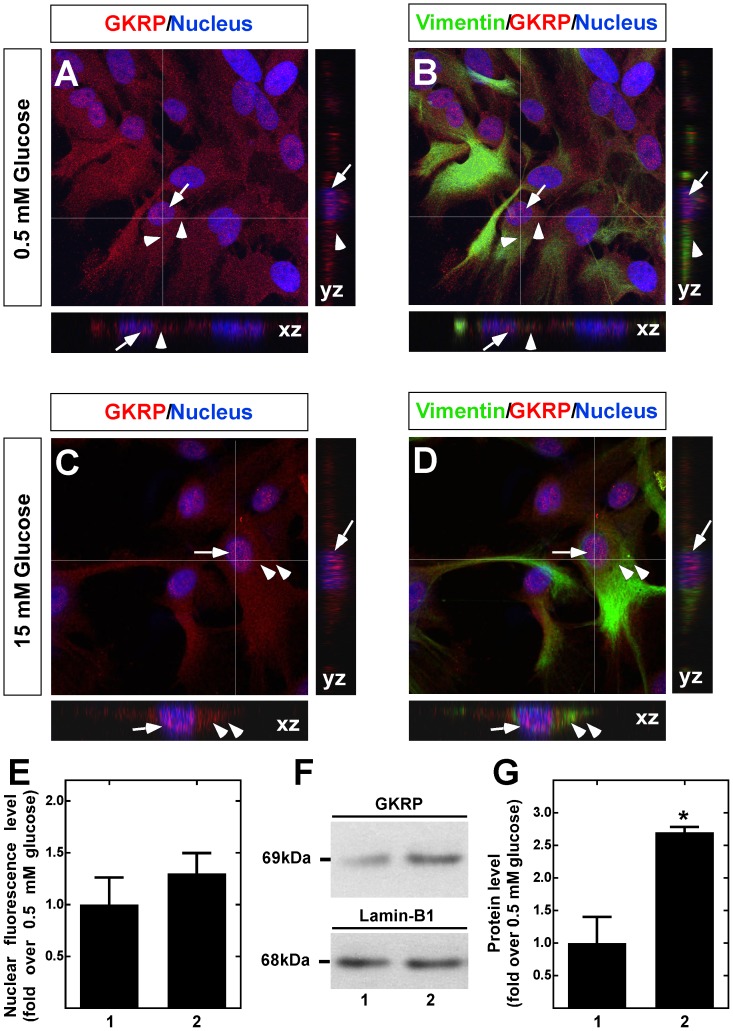
Dynamic localization of GKRP in cultured tanycytes in response to glucose. **A**–**D**. GKRP immunolocalization (red) in tanycytes preincubated with 0.5 mM glucose for 6 h (A–B) or incubated with 15 mM glucose for 30 min (C–D). Nuclei (A–C) and Vimentin (B, D) are stained in blue and green, respectively. Arrows and arrowheads show nuclear and cytoplasmic GKRP localization, respectively. Images were obtained by Z-stack reconstruction, and white lines indicate orthogonal planes (XZ and YZ) shown in the lateral and lower panels. **E**, Nuclear intensity of GKRP immunofluorescence in tanycytes cultured in the presence of 0.5 mM (lane 1) and 15 mM (lane 2) glucose. The results represent the mean±sd of 200 cells from three independent primary cultures. **F**, Immunoblots of GKRP (69 kDa, upper panel) and the nuclear marker, lamin-B1 (68 kDa, lower panel), in nuclear extracts obtained from cells cultured with 0.5 mM (lane 1) and 15 mM (lane 2) glucose. **G**, GKRP nuclear expression levels assessed by western-blot were quantified and normalized with lamin-B for cells cultured with 0.5 mM (lane 1) and 15 mM (lane 2) glucose. The data shows the means±SD from six independent experiments. * p<0.05; ** p<0.01. Scale bar in A-D, 50 µm.

GKRP nucleocytoplasmic redistribution in response to glucose was also evaluated. At 0.5 mM glucose, GKRP showed both cytosolic and nuclear localization (Fig. 7A, B). At 15 mM glucose, the nuclear fluorescence intensity was slightly increased (Fig. 7C, D), which was shown after quantification of the nuclear fluorescence intensity (Fig. 7E). However, immunoblot analysis of nuclear extracts from tanycytes revealed a similar increase in GKRP nuclear translocation as that observed with GK (Fig. 7F, G). Similar results were obtained when GKRP was analyzed in cytosolic extracts (Fig. S3C).

## Discussion

GK is mainly expressed in hepatocytes and pancreatic β cells, but also in the brain [22], [26], [29], [56]. GK has a critical role in pancreatic glucosensing and controlling the metabolic flow of glucose in the liver [56]. Our previous studies have demonstrated GLUT2 localization in the proximal pole of the tanycytes [6] and GK principally in the nucleus [13], suggesting that these cells may be involved in methabolic glucosensing in the hypothalamus, and therefore could have a role in feeding behavior. This idea is reinforced by the fact that rats injected i.c.v. with glucosamine, an inhibitor of GK, have an increased dietary intake [57]. Tanycytes have been implicated in an indirect control of the anorexigenic neuronal activity by glucose [11], [58]. Here we confirmed our prior report in which GK was detected in tanycytes rather than hypothalamic neurons. Specifically, whereas GK was detected solely in the cytoplasm of ependymal cells, in tanycytes, GK was mainly detected in the nucleus [13]. This observation may be due to the presence of GKRP, which has been previously reported in rat and human brain tissues [26], [29], [42].

Because coupling between tanycytes and neurons could have a role in hypothalamic glucosensing through glucose metabolism, it is important to know if GK localization is modulated by this nutrient in tanycytes. Here, we have effectively induced fasting condition, normoglycemia and hyperglycemia in male adult rats. These conditions allowed us to evaluate the intracellular localization of GK in both the liver and hypothalamus in response to glycemic conditions in the same animals. *In situ* and *in vitro* studies have demonstrated that hepatic GK reversibly translocates between the nucleus and cytoplasm in response to increases in glucose [52], [53], which was similar to the response detected in the present work. Conversely, our immunohistochemistry and Western blots results in hypothalamic tissue show that tanycyte GK has an opposing response to glucose. While the cellular mechanisms mediating GK translocation in tanycytes are unknown, in hepatic tissue, it is clear that under hypoglycemic condition, GK is bound to GKRP in the nucleus and is catalytically inactive [32]. It has been assumed that the removal of GK from the cytoplasm may reduce the futile substrate cycle between glucose and glucose-6-phosphate [30]. Here, we report for first time tanycyte GKRP expression *in vitro.* However, the nuclear GK localization suggests the presence of this protein *in situ*. It is possible that GKRP could have a similar role in GK compartmentalization in tanycytes; however, clearly there are substantial metabolic differences between hepatocytes and tanycytes. For example, the presence of glucose-6 phosphatase mRNA has been detected in the hypothalamus [59] but not specifically in tanycytes. Moreover, the presence of glycogen has been demonstrated in Hamster tanycytes by electron microscopy [60]. Jetton et al. [39] demonstrated a similar intracellular distribution of GK and glycogen deposits in primary cultures of hepatocytes; however, our previous study of GK ultrastructural-localization failed to detect a similar co-distribution in tanycytes [13]. For this reason, its contribution in controlling the metabolic flow of glucose in tanycytes requires additional analysis.

In tanycytes cultures, we demonstrated the expression of both GK and GKRP using RT-PCR, Western blot analysis, and molecular cloning. The unusual nucleocytoplasmic redistribution observed in the hypothalamus could be attributed to the expression of different GK and GKRP isoforms. Several splicing variants have been reported in the liver, pancreas and pituitary with most of them having no known function [26], [31]. Here, sequence analysis revealed that tanycytes express the pancreatic GK isoform, which was previously reported in the hypothalamus [26], [28], [61]. Tanycyte GKRP had a 99% identity to that found in the liver; the amino acid substitutions identified are not required for GK interaction to date [62].

Our kinetic characterization of GK recombinant revealed that the enzyme is functional *in vitro*. The S_0.5_ obtained for GK was 10 mM, which was the same value as that obtained by Matschinsky et al. [55] in GK cloned from pancreatic β cells. They also reported cooperative kinetics with a n_H_ 1.65, which was very similar to that obtained in this study (i.e., 1.5). Recent studies have attributed the cooperativity observed in this monomeric enzyme to a ligand-induced slow transition (LIST), in this case governed by glucose [49], [63]. Our results indicate that GK is noncompetitively inhibited by GKRP *in vitro*, lowering the V_max_ of the reaction, and no significant differences were observed in the S_0.5_ when GKRP was present. We have also been able to determine the affinity with which this cooperative enzyme binds to its regulatory protein through a graph proposed by Segel for an enzyme of this type, displaying a K_i_ value of 0.21 mM, which is close to that reported for the liver complex [40]. Given these results and considering that the molecular mass of GKRP is greater than that of GK, it is more likely that the inhibition is non-competitive.

Alvarez et al. [42] provide evidence for an interaction of GK and GKRP in the hypothalamus through GST-pull-down assays. However, the GK-GKRP interaction has not been studied in tanycytes and the glucose effect was not evaluated. Here, changes in the localization of both proteins induced by glucose were assayed *in vitro.* We previously have reported that 15 mM glucose induced an increase in [Ca^2+^]_i_ levels in tanycyte cultures, which was attributed to the glycolytic metabolism of glucose and not oxidative phosphorylation [14]. Thus, we used 0.5 and 15 mM glucose concentrations to analyze the nucleocytoplasmic redistribution of GK and GKRP. Similar to our results obtained *in situ,* we detected an increase in GK nuclear localization at 15 mM glucose as compared to 0.5 mM glucose. GKRP had a distribution pattern similar to GK; however, both immunocytochemical and Western blot analyses revealed that the subcellular changes in response to glucose were more discrete than observed for GK. GKRP translocation from the nucleus to the cytoplasm is controversial. Some authors have observed its shuttling in hepatocytes at high glucose concentrations [34], [64]. Conversely, colocalization of GK and GKRP has been reported in the same hypothalamic neurons mainly in the cytoplasm [65]. The results of this study revealed that GKRP is mainly localized in the nucleus, but at low extracellular glucose concentration, it was possible to find both GKRP and GK in the cytoplasm of tanycytes.

According to our previous work [13], we postulated that nuclear compartmentalization of GK and GKRP might play a role in tanycyte glucosensing. With increased CSF glucose concentrations, tanycytes could uptake glucose through GLUT2 [6], metabolizing it via anaerobic glycolysis to generate lactate and ATP [14], [19]. GK is considered a glucosensor protein because it catalyzes the rate-limiting step of glycolytic metabolism [66]. The importance of GK in tanycytes was demonstrated after injection of the GK inhibitor, alloxan, into the third ventricle, which induced the destruction of tanycytes as well as impaired feeding behavior [67]. The high glycolytic flux in the tanycytes allows release of lactate, which can be incorporated into AN anorexigenic neurons using MCT2 [11] to generate an increase in the ATP/ADP ratio, neuronal depolarization and a satiety response. Compartmentalization of GK by GKRP observed at 30 min of glucose may act as a molecular switch to arrest cellular signaling in response to increased glucose in the hypothalamus. When GK is inhibited by i.c.v injection of its pharmacological inhibitor, the feeding behavior was increased [57]; reduced activity of GK also generates an increase in the counterregulatory response to insulin-induced hypoglycemia in rats [68], which is in agreement with the inhibition of lactate flux from the glia to neurons. However, there is some controversy regarding the mechanism of tanycyte response and its function [13]–[14]. We have reported that tanycytes in cultures are responsive to glucose. Increased glucose (2–10 mM) generates a high glycolytic flux, which leads to the release of ATP by Cx43 hemichannels, activating P2Y receptors and thereby increasing [Ca^2+^]_i_ at the expense of intracellular stores [14]. On the other hand, the increase in [Ca^2+^]_i_ in acute *in situ* application of glucose and nonmetabolizable analogs of glucose in α-tanycytes was demonstrated [15]. The differences observed could be due to the different subpopulations tested. Therefore, is possible that tanycytes could be involved in both metabolic and non-metabolic glucosensing.

## Supporting Information

Figure S1 Changes in blood and cerebrospinal fluid glucose levels induced by i.p. injection of glucose in rats. **A-B**, Individually boxed rats were cannulated into third ventricle. After one week of recovery, the rats were fasted for 16 h. At 9:00, the animals were anesthetized to inject them i.p. with 4 g/kg body weight glucose or saline. Glycemia and glycorrhachia were measured in anesthetized rats at 15, 30 and 45 min post glucose injection and immediately after injection of saline (0 min). **A**, Glycemia level measured in 2 µL of blood obtained from the tail vein using the Accu-chek system. **B**, Glycorrhachia level measured in 2 µL of CSF obtained of third ventricle using the Accu-chek system. The number of rats used is shown in each bar.
(TIF)

Figure S2 Immunocytochemistry characterization of cultured tanycytes.Tanycytes obtained from rat hypothalamus at 1-day postnatal and were cultured for 3 weeks with 5 mM glucose. (A–E) Representative confocal images revealed a positive reaction for vimentin (A, green), MCT1 (B, green), DARPP32 (C, green), GLUT2 (D, green), Kir6.1 (E, green), and GK (F, green). Representative confocal images were negative for GFAP (G), MAP2 (H) and neurofilament (I) expression. Nuclei were stained with TOPRO-3 (blue). Scale bar, 80 µm.
(TIF)

Figure S3 Immunoblots of GK and GKRP in cytosolic protein extracts in cultured tanycytes in response to glucose. **A**, Immunoblots of GK (52 kDa; upper panel), GKRP (69 kDa; middle panel) and the cytosolic marker, β-actin (43 kDa, lower panel), in cytosolic extracts obtained from cells preincubated 0.5 mM glucose for 6 h (line 1) and incubated 15 mM glucose for 30 min (line 2). **B**, Quantitative analysis of GK cytosolic expression relative to β-actin. **C**, Quantitative analysis of GKRP cytosolic expression relative to β-actin. The cytosolic localization of GK and GKRP decreased with extracellular glucose. Data represent the means ± SD from six independent determinations. * p<0.05; ** p<0.01. Scale bar, 50 µm.
(TIF)
