# Expression of Concern: Dynamic Localization of Glucokinase and Its Regulatory Protein in Hypothalamic Tanycytes

**DOI:** 10.1371/journal.pone.0318865

**Published:** 2025-01-30

**Authors:** The PLOS One Editors

After publication of this article [1], concerns were raised about Fig 3. Specifically, lane 3 of the GK panel in Fig 3B appears similar to lane 3 of the GKRP panel in Fig 3D when rotated 180 degrees.

The authors stated that an error was made during the preparation of the figure due to the high number of membranes used in this paper. The authors commented that the original data underlying this study are no longer available, but they provided data from a repeat of these experiments. The repeat data did not resolve the concerns.

The *PLOS One* Editors issue this Expression of Concern to inform readers that in the absence of the underlying data, the concerns with this article cannot be fully resolved and the results should be interpreted with caution.
