# Supplementary figures and images for: Dynamic Localization of Glucokinase and Its Regulatory Protein in Hypothalamic Tanycytes
Source: PLoS One. 2014 Apr 16;9(4):e94035. doi: 10.1371/journal.pone.0094035 (PMC3989220; doi:10.1371/journal.pone.0094035)

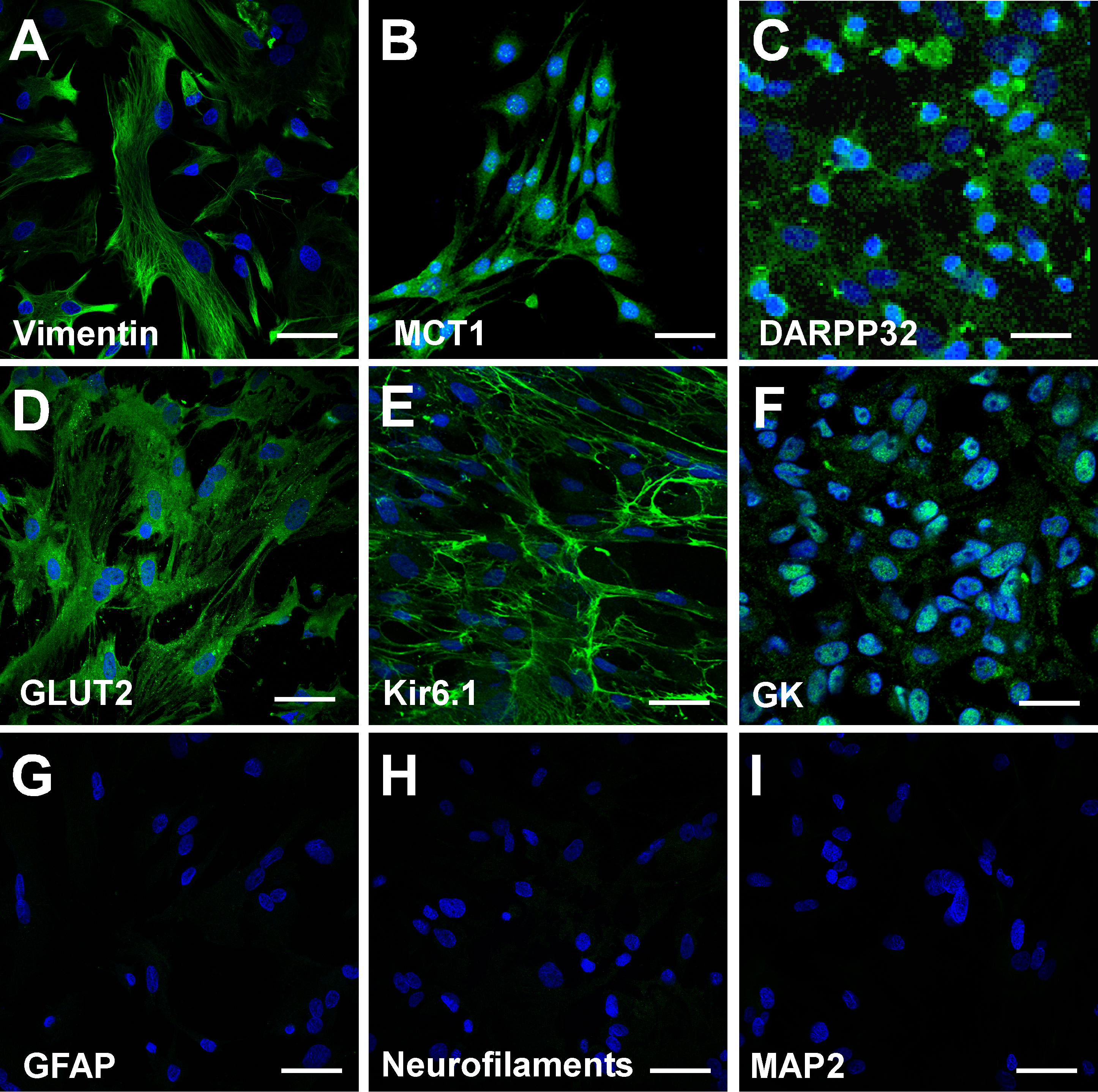

Supplement: Figure S2 — Immunocytochemistry characterization of cultured tanycytes.Tanycytes obtained from rat hypothalamus at 1-day postnatal and were cultured for 3 weeks with 5 mM glucose. (A–E) Representative confocal images revealed a positive reaction for vimentin (A, green), MCT1 (B, green), DARPP32 (C, green), GLUT2 (D, green), Kir6.1 (E, green), and GK (F, green). Representative confocal images were negative for GFAP (G), MAP2 (H) and neurofilament (I) expression. Nuclei were stained with TOPRO-3 (blue). Scale bar, 80 µm. (TIF) [file pone.0094035.s002.tif]

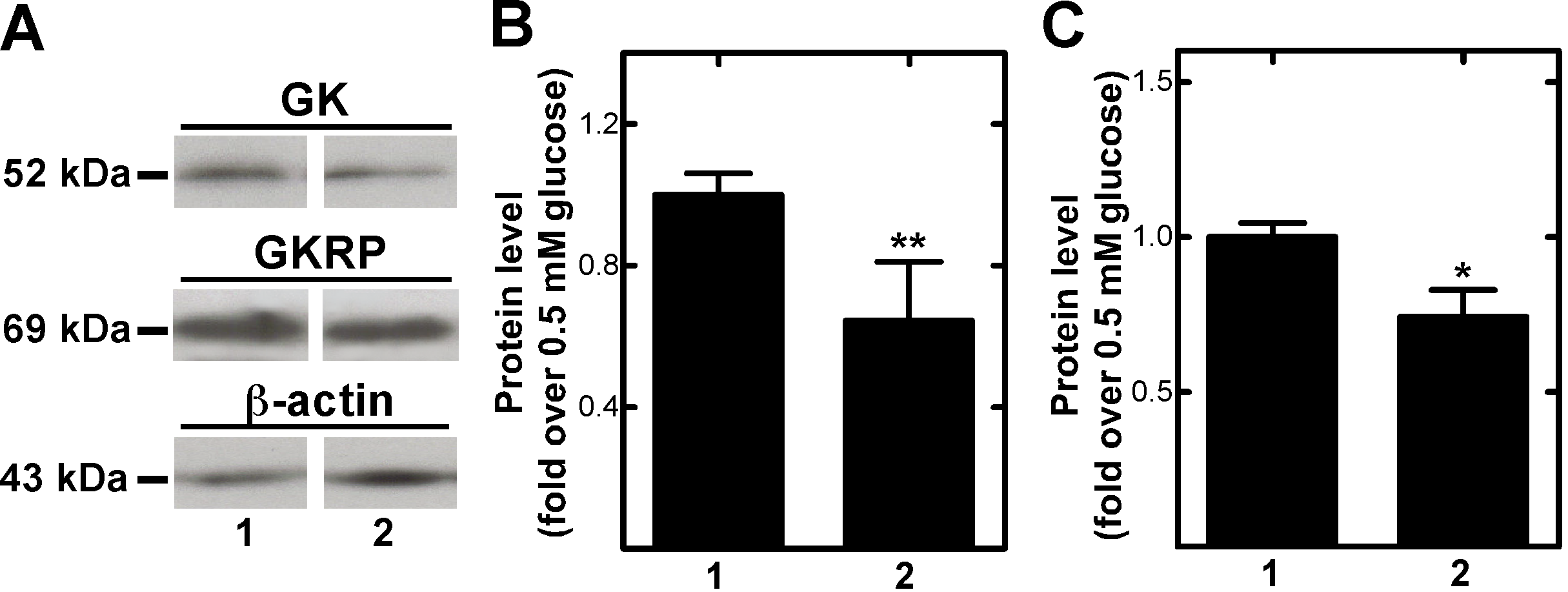

Supplement: Figure S3 — Immunoblots of GK and GKRP in cytosolic protein extracts in cultured tanycytes in response to glucose. A, Immunoblots of GK (52 kDa; upper panel), GKRP (69 kDa; middle panel) and the cytosolic marker, β-actin (43 kDa, lower panel), in cytosolic extracts obtained from cells preincubated 0.5 mM glucose for 6 h (line 1) and incubated 15 mM glucose for 30 min (line 2). B, Quantitative analysis of GK cytosolic expression relative to β-actin. C, Quantitative analysis of GKRP cytosolic expression relative to β-actin. The cytosolic localization of GK and GKRP decreased with extracellular glucose. Data represent the means ± SD from six independent determinations. * p<0.05; ** p<0.01. Scale bar, 50 µm. (TIF) [file pone.0094035.s003.tif]
